# SADI, SHARE, and the *in silico* scientific method

**DOI:** 10.1186/1471-2105-11-S12-S7

**Published:** 2010-12-21

**Authors:** Mark D Wilkinson, Luke McCarthy, Benjamin Vandervalk, David Withers, Edward Kawas, Soroush Samadian

**Affiliations:** 1 Heart + Lung Institute at St. Paul’s Hospital, University of British Columbia, Vancouver, BC, Canada, V6Z 1Y6

## Abstract

**Background:**

The emergence and uptake of Semantic Web technologies by the Life Sciences provides exciting opportunities for exploring novel ways to conduct *in silico* science. Web Service Workflows are already becoming first-class objects in “the new way”, and serve as explicit, shareable, referenceable representations of how an experiment was done. In turn, Semantic Web Service projects aim to facilitate workflow construction by biological domain-experts such that workflows can be edited, re-purposed, and re-published by non-informaticians. However the aspects of the scientific method relating to explicit discourse, disagreement, and hypothesis generation have remained relatively impervious to new technologies.

**Results:**

Here we present SADI and SHARE - a novel Semantic Web Service framework, and a reference implementation of its client libraries. Together, SADI and SHARE allow the semi- or fully-automatic discovery and pipelining of Semantic Web Services in response to *ad hoc* user queries.

**Conclusions:**

The semantic behaviours exhibited by SADI and SHARE extend the functionalities provided by Description Logic Reasoners such that novel assertions can be automatically added to a data-set without logical reasoning, but rather by analytical or annotative services. This behaviour might be applied to achieve the “semantification” of those aspects of the *in silico* scientific method that are not yet supported by Semantic Web technologies. We support this suggestion using an example in the clinical research space.

## Background

Clarity is the cornerstone of Science. In the tradition of the formal scientific method, experiments should be explicit and thorough in describing every stage of the analysis, starting with the initial question or hypothesis, continuing on through the methodology by which candidate data were selected and analyzed, and finishing with a fully-documented result, including all provenance information (which resource, which version, when, and why). As modern biology becomes increasingly *in silico*-based, many of these best practices are being managed with much higher efficiency. The emergence of Web Services and analytical workflows as first-class referenceable and shareable objects in bioinformatics [1,2] has led to a high level of precision in describing *in silico* “materials and methods”, as well as the ability to automate the collection of highly detailed provenance information. However, earlier stages in the scientific process - the posing of the hypothesis and the selection of candidate data - are still largely limited to human cognition; we typically pose our hypotheses in the form of sentences, and we often select and screen candidate data based on expert knowledge or intuition.

Recently, new standards have emerged that allow us to explicitly express “Knowledge”. In particular, the endorsement of the Web Ontology Language (OWL [3]) by the World Wide Web Consortium has provided a global standard for knowledge representation which is showing particularly rapid adoption within the life sciences and health sciences communities [4]. Though there are numerous examples [5] of ontologies being used to describe “what is” (i.e. to describe particular aspects of biological reality), we have found no examples of ontologies being used, in practice, to describe “what might be” (i.e. a hypothetical, unproven view of biological reality). Given the constantly changing nature of “biological reality”, we find this distinction to be somewhat artificial - we would argue that ontologies, in general, can and usually do represent hypotheses. If this were true, the logical constructs that exist in the OWL Description Logic (OWL-DL) might allow these ontologies/hypotheses to be explicitly expressed at a level of detail and granularity sufficient to make them the *in silico* equivalent to a scientific hypothesis expressed in natural language.

Hypotheses encoded in OWL would have a number of significant advantages over hypotheses represented in natural language; they would be unambiguous, extensible by third parties, and could be tested computationally. These features would make such OWL constructs an excellent platform for scientific discourse and disagreement. However, given that OWL reasoners are currently only able to compute inferences for a single, locally stored dataset, the testing and comparison of such hypotheses would be constrained by the human labour of gathering and integrating data from many sources.

SADI - Semantic Automated Discovery and Integration [6] - is a set of “best-practices” for modeling Semantic Web Services in the scientific domain, and an open-source set of code modules in Perl and Java that adhere to these best-practices. SHARE - the Semantic Health And Research Environment [7] - is a prototype client that uses the SADI Framework to demonstrate how applications might take advantage of the semantic features of SADI Web Services. SHARE, augments OWL reasoners with the ability to retrieve entities from remote data sources at the time of reasoning, and to validate relationships between those entities using arbitrary computational tools. Here we provide a brief progress report for the SADI and SHARE projects. We then discuss how SHARE enables the creation and testing of scientific hypotheses without dependency on locally installed data and software. We believe that, by encouraging the explicit encoding, sharing, comparing, and editing of ideas among researchers in the community, SHARE reveals the plausibility of engaging in a novel form of highly detailed scientific discourse, currently lacking in the *in silico* scientific process. We then speculate, using demonstrative queries and ontologies, how SADI and SHARE might contribute to the vision of the complete *in silico* scientific method described above.

## SADI update

SADI Semantic Web Services are distinct from traditional Web Service frameworks in that W3C Semantic Web technologies Resource Description Framework (RDF) [8] and OWL are used at every level of the Web Service “stack”. Service interfaces are defined in OWL-DL, consisting of two classes representing the service input, and output respectively. Services consume OWL Individuals of the input class, and return OWL Individuals of the output class. The key best-practice mandated by the SADI framework is that the URI of the input and output individuals must be the same. As such, every service becomes an ‘annotation’ service, where the data input to a service, and the data generated by the service execution, are explicitly linked by a meaningful set of RDF predicates. Since almost all Services in the bioinformatics domain are stateless and atomic, this restriction is not significant, and to date we have not encountered a bioinformatics Web Service that could not be modeled in SADI. The URI-best-practice also makes it possible to automatically determine what a service does by simply comparing the input and output classes. The predicates added, input and output data-types for all SADI services are automatically indexed and made available for searches in a publicly accessible SPARQL [9] endpoint.

A variety of SADI-compliant tools are available from both the client and service-provider perspectives. For deploying SADI services in Java, a codebase is available on Google Code [10]. The Java codebase uses Maven for dependency management and a skeleton Eclipse project can be downloaded to make the process of service development as painless as possible. For service providers in Perl, a code module - OWL2Perl [11] - is available in CPAN that consumes OWL class definitions and creates Perl code modules that facilitate the creation of OWL Individuals representing those classes. Thus, the parsing of input data, and creation of output data, for any given SADI service in Perl is greatly simplified. To simplify things further, a plug-in [12] has been written for the Protégé [13,14] ontology editing environment that semi-automates the creation of SADI Services simply by dragging and dropping ontology nodes from the Protégé canvas onto the SADI canvas, and providing a few simple annotations. Service code is written (either in Java or in Perl) and the provider simply needs to add their business-logic and fill-in the stub-values provided in order to create a functional service. Moreover, services can be tested by creating an OWL Individual inside of Protégé and sending that data to the service. In future iterations, these testing input and output data will be captured by the system and used for daily “unit-tests”, which could be used to provide QoS statistics or automatically alert service providers that a service has stopped functioning normally. Two SADI client tools are available. SHARE (discussed below) and a SADI plug-in [15] to the Taverna [16] workflow editing and enactment environment. The SADI Taverna plug-in enables semantically-enhanced searches for SADI Web Services, such that, at any given point in the workflow, services can be discovered that feed-into, or consume, data-types appropriate to the currently selected service. This greatly simplifies construction of valid workflows. The SADI plugin also creates RDF-formatted data from non-SADI Web Services, and/or extracts data from RDF in order to pass it on to downstream non-SADI Web Services, thus SADI services can interact seamlessly with traditional Web Services in the Taverna environment.

## SHARE update

SHARE exposes SADI Web Services as if they were a virtual, distributed SPARQL endpoint. It consumes SPARQL queries and deconstructs them to individual triple-patterns, then maps the predicates and data-types required to SADI services capable of creating those data. A workflow of Services is automatically designed, data is passed/generated during execution of that workflow, and the final transient database is then used to resolve the original query. SHARE can also deconstruct OWL-DL class definitions referred-to in a SPARQL query, and similarly map the property-restrictions in those classes to a workflow of SADI services capable of generating the properties defined in the OWL class. Thus, OWL Individuals representing arbitrary OWL classes can be dynamically discovered or generated from distributed data and analytical tools. The SHARE client can be configured to use any OWL reasoner that exposes an interface compatible with the Jena Semantic Web Framework.

## SADI and SHARE and the *in silico* scientific method

A demonstrative query will reveal the novel features of the SADI + SHARE system that, we believe, provide insight into how explicit hypothesis specification and automated hypothesis evaluation might work in a Semantic Web environment. The query below retrieves the latest Blood Urea Nitrogen and Creatinine blood chemistry levels from patients who are likely to be rejecting their kidney transplants (this query can be run from the SHARE client at http://dev.biordf.net/cardioSHARE/, which uses the Pellet 2 OWL reasoner [17])

PREFIX rdf: <http://www.w3.org/1999/02/22-rdf-syntax-ns#>

PREFIX patients: <http://sadiframework.org/ontologies/patients.owl#>

PREFIX pred: <http://sadiframework.org/ontologies/predicates.owl#>

SELECT ?patient ?bun ?creat

FROM <http://sadiframework.org/ontologies/patients.rdf>

WHERE {

?patient rdf:type patients:LikelyRejecter.

?patient pred:latestBUN ?bun.

?patient pred:latestCreatinine ?creat.

}

Of particular relevance in this query is the constraint that the patient should be of type ‘Likely Rejecter’. Examining the OWL definition of Likely Rejecter, we find that Likely Rejecters have a collection of blood creatinine levels that are ‘elevated’. Elevated creatinine levels are defined as a collection of measurements that have an ‘increasing’ linear regression model. Linear regression models have features such as slope, and intercepts. The patients.rdf database contains patients who each have a time-course of various blood chemistry measurements.

When SHARE finds the [patient type Likely Rejector] clause, it examines what data exists in the database, examines the definition of Likely Rejecter, and then synthesizes a workflow capable of determining which patients fulfil the Likely Rejecter class definition. This includes discovery and execution of a SADI Web Service that can do a linear regression analysis on X-Y coordinate data, and the automated detection (by semantic reasoning) that a time-course of blood chemistry measurements are simply a specialized type of X-Y coordinate data. Once the analysis is complete, the Pellet2 reasoner is used to classify patients as Likely Rejecter (or not), and the remainder of the query is resolved for those patients (also using Web Services that map to the latestBUN and latestCreatinine predicates).

During resolution of this query, the Likely Rejecter OWL class definition acted as an abstract workflow. Concretization of that workflow happened dynamically at run-time by (a) examining the “needs” of the Class, then (b) determining which of those “needs” existed in the dataset, which were purely logical constructs that could be managed by the reasoner, and which required mapping onto SADI services capable of doing database look-ups or analytical operations to fulfill those needs. Importantly, the individual components of the Likely Rejecter class are granular and largely “non-controversial” (e.g. that x-y coordinate data can be represented as a linear regression,and that linear regression models have a slope); however the Likely Rejecter class itself is controversial - when faced with this definition, Clinicians will often complain, for example, that that creatinine levels do not have to be increasing in order to be dangerously elevated. As such, Likely Rejecter represents a category, assembled from distributed concepts and relationships, that represents one perspective of what defines a likely transplant rejecter. Effectively, it is a hypothetical class of patients, and individuals that fit the hypothesis (if any) are determined through an automatically generated analytical pipeline.

## Other observations

Some aspects of the SADI + SHARE behaviour are quite distinct from the current state-of-the-art in Semantic Web infrastructure. With existing OWL/RDF frameworks, a DL reasoner is provided with an ontology and putative instance data. The reasoner examines the data, creates new assertions within that dataset based on the logical axioms in the ontology, and then classifies the instances into the various ontological categories. With SHARE, an ontology and putative instance data are provided to the query engine, and new assertions are created in the dataset through both DL reasoning, and the discovery of SADI Web Services capable of adding the assertions defined in the ontology. As such, the system extends our ability to automatically classify instance data beyond what current DL reasoners can achieve, because the SHARE+SADI system can add new assertions that are not derived from pure logic, but rather by expert-knowledge encoded as Web Services. Thus, perhaps the most important consequence of SADI+SHARE is that the motivation to encode the knowledge of the “professional annotator” is significantly enhanced. Currently, in the typical life sciences semantic framework, knowledge about how to interpret the data is encoded in ontologies and shared; however, knowledge about the process of creating the data - annotation - is contained in a small set of experts who represent a specific community or institutional agreement [18]. In order to apply a given annotation to a piece of data (i.e. a predicate, in Semantic Web terms) the data goes through the institutional process and thereby becomes annotated. In SADI + SHARE, not only is the institutional annotation process encoded in the form of a Web Service (which in itself is not novel), but the annotation process is linked to the interpretive knowledge layer; the desired knowledge drives the automated discovery of the annotations that are needed, as well as driving that annotation to be executed over one’s local, personal data set. There are already numerous pragmatic reasons to encode frequently-applied algorithms or repetitive tasks; however, given the scenario just described, the incentive to encode institutional/curatorial knowledge into Web Services becomes, in our opinion, even more compelling!

As a direct consequence of reducing the need for professional annotators, we simultaneously reduce the need for community agreement in general. The Semantic Web in healthcare and life sciences currently houses numerous ontology consortia whose primary purpose is to define consortia-approved ontological classes. The message to these consortia from observing the behaviours of SADI + SHARE may be that it is the predicates, rather than the classes, that we need agreement on. In fact, if we entertain the possibility raised above that ontological classes represent hypotheses, then we might *hope for* community disagreement about these ontological classes, in order to drive new scientific discovery.

## Conclusion

The ability to automatically find instances of hypothetical classes makes it feasible and useful to now consider new opportunities to support the explicit construction, sharing, and comparison of hypotheses - a formalization of the traditional “discourse and disagreement” components of the scientific method. As an increasing number of predicates become available through the SADI Semantic Web Service framework, increasingly complex hypotheses will be able to undergo automatic resolution using tools like SHARE.

## List of Abbreviations

CPAN: Comprehensive Perl Archive Network; DL: Description Logic; OWL: Web Ontology Language; QoS: Quality of Service; RDF: Resource Description Framework; SADI: Semantic Automated Discovery and Integration; SHARE: Semantic Health and Research Environment; URI: Uniform Resource Identifier

## Author’s Contributions

MDW conceived of the SADI project and wrote the manuscript. LM and BV conceived of and wrote the SHARE client system and the libraries that enable the decomposition of OWL classes into a Web Service workflow. DW wrote the plugin to make SADI Services accessible from Taverna. EK wrote the plugin that allows semi-automated writing and testing of SADI Web Services from within Protégé, and SS wrote the cardiovascular and clinical ontology,and implemented several of the SADI services that make the Likely Rejecter example query, among others, possible.
